# Key features of the innate immune response is mediated by the immunoproteasome in microglia

**DOI:** 10.1038/s41598-025-25341-5

**Published:** 2025-11-21

**Authors:** Salman Izadjoo, Kasey E. Moritz, Guzal Khayrullina, Elizabeth M. Bergman, Brendan M. Melvin, Matthew W. Stinson, Summer G. Paulson, Nikki M. McCormack, Kelsey N. Anderson, Lunndon A. Lewis, Jeremy D. Rotty, Barrington G. Burnett

**Affiliations:** 1 Neuroscience Program, F. Edward Hebert School of Medicine, Uniformed Services University, 4301 Jones Bridge Rd, Bethesda, MD 20814 USA; 2 Molecular and Cell Biology Program, Uniformed Services University, F. Edward Hebert School of Medicine, Bethesda, MD USA; 3 Center for Neuroscience and Regenerative Medicine, Uniformed Services University, Bethesda, MD USA; 4 Department of Anatomy, Physiology and Genetics, Uniformed Services University, Bethesda, MD USA; 5 Uniformed Services University Research Training Program, University of Maryland, College Park, MD USA; 6 Department of Biochemistry, Uniformed Services University, Bethesda, MD USA

**Keywords:** Immunoproteasome, Microglia, Innate immunity, Complement, Cytokines, NFκ-B, Phagocytosis, ONX-0914, Cell biology, Immunology, Neuroscience, Neurological disorders

## Abstract

Microglia are the resident immune cells of the central nervous system (CNS). We and others have shown that the inflammatory response of microglia is partially regulated by the immunoproteasome, an inducible form of the proteasome responsible for the generation of major histocompatibility complex (MHC) class I epitopes. While the role of the proteasome in the adaptive immune system is well established, emerging evidence suggests the immunoproteasome may have discrete functions in the innate immune response. Here, we show that inhibiting the immunoproteasome reduces the IFNγ-dependent induction of complement activator C1q, suppresses phagocytosis, and alters the cytokine expression profile in a microglial cell line (BV2) and microglia derived from human inducible pluripotent stem cells. Moreover, we show that the immunoproteasome regulates the degradation of IκBα, a modulator of NF-κB signaling. Finally, we demonstrate that NADH prevents induction of the immunoproteasome, representing a potential pathway to suppress immunoproteasome-dependent immune responses.

**Supplementary Information:**

The online version contains supplementary material available at 10.1038/s41598-025-25341-5.

## Introduction

As immune cells of the central nervous system (CNS), microglia constantly survey the brain and spinal cord for changes to the local environment. In response to CNS infection or injury, microglia transition to a reactive state, where they are responsible for mounting an immune response. The adaptive immune response occurs over a period of days to weeks and is more specific to particular pathogens or invaders. The innate immune response in the CNS is primarily mediated by microglia and is activated within hours of pathogen recognition (For review^1^). There are three major components of the innate immune response. First, activation of the complement cascade activates additional immune cells, facilitating identification of bacteria, dying cells and foreign pathogens. The complement system can also target invading cells for destruction. Second, dead cells, foreign particles and cell debris are cleared by phagocytosis. Finally, the innate immune response involves production of cytokines and chemokines which act to promote cell migration and recruitment of additional immune cells to the site of injury or infection.

Activation of microglia and the subsequent innate immune response is critical for defense against infection, however, during trauma, it can have confounding effects. The inflammatory response is important for returning the CNS to homeostasis, however increased microglia reactivity and the subsequent inflammatory response can result in reduced functional recovery following trauma and stroke. In addition, persistent activation of the immune response can result in an exacerbated response to subsequent injuries. Decreasing inflammation following trauma can reduce some of the long-term functional deficits associated with CNS injury. Furthermore, neuroinflammation has been linked to neurodegenerative disease progression such as in Alzheimer’s and Parkinson’s diseases^2,3^. Thus, controlling the inflammatory response of microglia following CNS disruption could prove to be a beneficial therapeutic option.

Protein degradation machinery lies at the intersection of cellular homeostasis and neuroinflammation. The proteasome is the final destination for proteins tagged for degradation by the ubiquitin proteasome system^4^. During trauma, infection and subsequent neuroinflammation, the constitutive proteasome subunits are replaced by immunoproteasome subunits^5,6^. The inducible immunoproteasome has alternative regulator and catalytic subunits than the constitutive proteasome (For review^7^). IFNγ, a pro-inflammatory cytokine up-regulated during CNS trauma triggers the formation of the immunoproteasome. We have previously reported that inhibiting the immunoproteasome alters the microglia transcriptome profile, most notably in regards to the immune response and inflammation^6^. While the immunoproteasome has been shown to be critical in peptide processing for antigen presentation during the adaptive immune response, there have been few studies to date that link the immunoproteasome and the innate immune response in microglia^7,8^. Indeed, using both mouse and human microglia models, it was demonstrated that immunoproteasome deficiency disrupts cellular protein homeostasis, leading to stress responses and alterations in the ubiquitin-modified proteome. This disruption affects various microglial functions, including the regulation of innate immune signaling, phagocytosis, and cytokine production, highlighting the importance of immunoproteasomes in maintaining microglial health and function^8^.

We thus sought to examine the role of the immunoproteasome on the innate immune response of microglia and investigate the mechanism of immunoproteasome mediation of inflammation. Our study demonstrates that pharmacological and genetic ablation of the immunoproteasome subunit β5i reduces IFNγ-dependent expression of the classical complement system activator C1q, phagocytosis, and chemokine production. In addition, we demonstrate that genetic ablation of β5i results in reduced IκBα degradation, signifying increased NF-κB activation, providing insight into a possible mechanism of immunoproteasome-mediated inflammatory response in microglia. Finally, we show that NADH, a known inhibitor of microglia-mediated neuroinflammation, blocks IFNγ-mediated induction of the immunoproteasome. Taken together, this study provides initial evidence that the immunoproteasome mediates key features of the innate immune response in mouse and human microglia exposed to IFNγ.

## Materials and methods

### Cell culture

BV-2 cells (A gift from Dr. Kimberly Byrnes) data a were cultured under standard conditions in DMEM (Thermofisher) medium containing 5% FBS (Sigma), 2 mM L-Glutamine, 100 units/ml penicillin and 100 µg/ml streptomycin (Thermofisher). Cells were passaged 2–3 times per week. CRISPR/Cas9-mediated β5i knock-out BV-2 cells were created as previously described^6^. We obtained doxycycline inducible microglia IPSCs from Dr. Michael Ward’s lab^9^^10^. IPSC lines were maintained in standard 6-well tissue culture plates coated with growth factor reduced Matrigel (Cat. 354277, Corning) diluted 1:100 in DMEM/F12 (Gibco) on the same day as iPSC plating. Frozen stocks of iPSCs were thawed and plated on Matrigel-coated plates in Essential 8™ Basal Medium (Gibco) supplemented with 10µM ROCK inhibitor (Y-27632 dihydrochloride; Tocris #1254). iPSC lines were passaged using 0.5 mM EDTA in PBS without CaCl_2_ and MgCl_2_ (Life Technologies). Cells were maintained in incubators at 37 °C, 5% CO_2_. The iPSCs and differentiated cells were confirmed to be mycoplasma negative.

β5i Knock-out BV-2 Cell Line: BV-2 cells were seeded to 70–80% confluence, then transfected with β5i double nickase plasmid containing GFP and Puromycin selection markers per manufacturer instructions (sc-421450-nic, Santa Cruz Biotechnology). 24 h following transfection, Puromycin (10 µg/ml) was added to the cells and maintained for 3 d. Following, cells were expanded in the presence of 2 µg/ml for 1–2 weeks and western blots were performed to validate the absence of β5i protein.

### IPSC microglia differentiation

We obtained doxycycline inducible microglia IPSCs from Dr. Michael Ward’s lab (Dräger et al., 2021). Doxycycline-inducible microglia iPSCs were grown in StemFlex or Essential 8™ Basal Medium (Gibco) until reaching at least 50% confluency and were grown for at least 24 h without ROCK inhibitor (Tocris #1254). Cells were dissociated, centrifuged and pelleted cells were resuspended in Day 0 differentiation medium containing the following: Essential 8™ Basal Medium as a base, 10µM ROCK inhibitor, and 2 µg/ml Doxycycline (Clontech). Doxycycline inducible microglia iPSCs were counted and seeded onto double coated plates (Poly-D-Lysine-precoated Bio plates (Corning) + Matrigel) with 350,000 cells per /well for a 6-well plate. Media was replaced on day 2 with differentiation media consisting of Advanced DMEM/F12 Medium (Gibco) as a base medium, 100X GlutaMAX™ (Gibco), 2 µg/ml doxycycline, 100 ng/ml Human IL34 (Peprotech) and 10 ng/ml Human GM-CSF (Peprotech). On day 4, the medium was replaced with maintenance Microglia medium, containing Advanced DMEM/F12 as a base medium combined with 100X Antibiotic-Antimycotic (1X Anti-Anti) (Gibco), 100X GlutaMAX, 2 µg/ml doxycycline, 100 ng/ml Human IL-34 and 10 ng/ml Human GM-CSF, 50 ng/ml Human M-CSF (Peprotech) and 50 ng/ml Human TGFB1 (Peprotech), and 50 μm Mevalonate (Sigma). On Day 8, half the media was replaced with fresh Microglia medium. Microglia were cultured for up to 12 additional days in maintenance Microglia medium with full medium changes every 3–4 days. Cells were assayed on day 9.

### Cytokine and drug treatment

BV-2 cells were plated 60–70% confluent, allowed to adhere overnight then treated with 200 U/mL of IFNγ (R&D Systems) for 24 h. Immunoproteasome inhibition was achieved using 100 nM ONX-0914 (UBPBio). Cells requiring nicotinamide adenine dinucleotide treatment were pre-treated for 24 h (100 µM, Sigma-Aldrich).

### RNA isolation and gene expression analysis

Gene expression analysis was performed as previously described^6^. Briefly, RNA was isolated by the Trizol and chloroform extraction method, then purified RNA was then converted to cDNA using a Veriti thermal cycler and a high capacity cDNA conversion kit (Applied Biosystems). Gene levels were measured using pre-validated Taqman probes (Life Technologies).

### Western and native gel electrophoresis

For western blotting, cells were lysed (20 mM Hepes, 0.32 M Sucrose, 5 mM MgCl_2_, 2 mM ATP, 0.2% W/V NP-40, 2 mM DTT, Protease Inhibitor, pH 7.2), combined with sample buffer and 10 µg total protein run on 4–12% gels (Invitrogen). All antibodies were obtained from Abcam unless otherwise stated: Psmb5 (ab3330), Psmb8 (ab3329), IκBα (sc371, Santa Cruz), β actin (A3854, Sigma), C1q (ab71089), IP-10 (MAB466, R&D Systems), CCL2 (NBP1-07035, Novus Biologicals).

### NF-κB activation assay

The activation of NF-κB pathway was measured through transcription factor subunits in the nuclear fraction using NF-κB Activity Assay Kit 2 (Fivephoton Biochemicals, 2010). Nuclear and cytoplasmic fractions were isolated from BV-2 cells 24 h post-treatment per manufacturer’s protocol, and subjected to Western blot analysis (*n* = 4 per group) (Invitrogen, Carlsbad, CA). PVDF membrane was probed for p65 (1:400, Fivephoton). Bands were quantified using Image J densitometry and normalized to GAPDH (1:2000).

### Flow cytometry

Cells were prepared for flow cytometry as previously described [Reference 6, if applicable], with modifications. Briefly, cells were rinsed twice with warm phosphate-buffered saline (PBS) to remove residual media. Cells were then dislodged from the culture surface by scraping in fluorescence-activated cell sorting (FACS) buffer (e.g., 1X PBS, 1% bovine serum albumin (BSA), 0.1% sodium azide; ThermoFisher). The cell suspension was transferred to 1.5 mL microcentrifuge tubes and centrifuged at 1400 RPM (approximately 300 x g) at room temperature for 5 min to pellet the cells. The supernatant was carefully aspirated, and the cell pellet was resuspended in 100 µL of FACS buffer.

For surface staining, cells were incubated with pre-conjugated antibodies from Biolegend: anti-CD21/35 (PE-anti-mouse CD21/35, BL 123410; clone 7E9; 1:100 dilution), anti-CD11b (PE anti-mouse/human CD11b, BL 101208; clone M1/70; 1:100 dilution), anti-CD88 (PE-anti-mouse CD88, BL 135806; clone 20/70; 1:100 dilution), and anti-CD93 (PE-anti-mouse CD93, BL 336108; clone VIMD2, 1:100 dilution). The antibody concentrations were used according to the manufacturer’s recommendations. The cell suspensions were incubated with the antibodies for 15 min at 4 °C in the dark to allow for optimal antibody binding. Following incubation, cells were washed twice with 1 mL of FACS buffer to remove unbound antibodies. After the final wash, cells were resuspended in 200 µL of FACS buffer for analysis. Samples were a read in the PE channel using a BD Accuri C6 flow cytometer, and data analysis was performed using FlowJo™ software version 10.7.1 (Becton, Dickinson and Company).

### Phagocytosis assay

BL-21 DE3* (ThermoFisher) were transformed with pAcGFP1 vector as described previously^11^. 40 µl of bacteria (3.0 × 10^8^) were combined with 1 ml of culture media and added to each well of BV-2 cells which had been pre-treated with 200 U/mL IFN γ and/or 100nM ONX-0914 for 24 h. Cells were incubated at 37 °C for 30 min then washed 3 times with ice-cold PBS before being dislodged by scraping with FACS buffer and submitted to flow cytometry (BD Accuri C6, BD Biosciences). Cells were gated so that only viable cells were counted. 10,000 cells per treatment group were counted. To adjust for background, control cells that were not incubated with E. coli were used for each experiment (*n* = 6). The percentage of GFP positive cells was measured and data were analyzed using an ANOVA with Tukey’s *post hoc* analysis.

Bead-based phagocytosis was assessed using a bead-based assay. IgG was first labeled with pHrodo™ iFL Red following the manufacturer’s instructions (ThermoFisher Scientific, Cat# P36014). Subsequently, 60 µg of labeled IgG was opsonized with 60 µg of 2 μm Polybead Carboxylate Microspheres in 200 µL of 1X PBS. The IgG-opsonized beads were then vigorously vortexed. Cells were seeded into a 24-well plate at a desired density. Following cell attachment, 4 µL of the opsonized beads were added to each well and the plate was gently shaken to ensure even distribution. The phagocytosis assay was then run on a Cytation 5 Imaging Reader (Gen5 Image Prime version 3.11; https://www.agilent.com/en/product/cell-analysis/cell-imaging-microscopy/cell-imaging-microscopy-software/biotek-gen5-software-for-imaging-microscopy-1623226). Images were captured every 15 min for 2 h using both Relief Contrast and TRITC channels. Four positions within each well were imaged. After the 2-hour incubation, the media was aspirated from the wells. Cells were then stained with a 1:10,000 dilution of Hoechst stain (1.2 µg/mL final concentration) in 1X PBS for 5 min. Following thorough washing with PBS, a final image was captured using Relief Contrast, TRITC, and DAPI channels.

Quantification was performed using the Cytation 5 software. The software was used to count the number of cells in the DAPI channel and to measure the number, size, and intensity of phagosome containing beads in the TRITC channel. Data analysis was then performed using the software outputs. The ratio of phagosome containing beads to cell number, the difference in phagosome containing bead intensity per condition, and the difference in average area per condition were all calculated.

### Cytokine panel

The Proteome Profiler Mouse Cytokine Array Panel Cytokine expression was used to measure cytokine expression at 24 h post-treatment (R& D systems, Minneapolis, MN). The assay was conducted per the manufacturer’s instructions. 300 µg of protein was collected from cell lysates (*n* = 4 per group). HLImage + + machine vision and image processing software with Array QuickSpots (version 25.6.0.1) was used to analyze the cytokine expression density (2011 Western Vision Software). Duplicate spots representing individual cytokines were averaged and background was subtracted. For statistical analysis, all spots that were not detectable were counted as 0. Data are normalized to the IFNγ treatment group.

### Data analysis

All data were analyzed using SPSS24 for Windows. Outliers were removed by calculating the Z-score (95% confidence interval) for each data set. Data points where Z > 2 were considered outliers and omitted from analysis. For analysis between 2 groups (Control and IFNγ, sham and injury) an independent samples t-test (2-tailed) was conducted. Levene’s test was used to assess variance and normality and appropriate transformations were applied when necessary. For experiments involving multiple treatment groups, one-way analysis of variance was used (ANOVA), with Tukey’s HSD *post-hoc* to compare differences between groups when appropriate. For all figures, *p < .05, **p < .01, ***p < .001, ****p < .0001. Data are presented as mean values ± standard error of the mean (SEM).

## Results

### Immunoproteasome Inhibition decreases IFNγ-dependent activation of the complement cascade

**Fig. 1:**
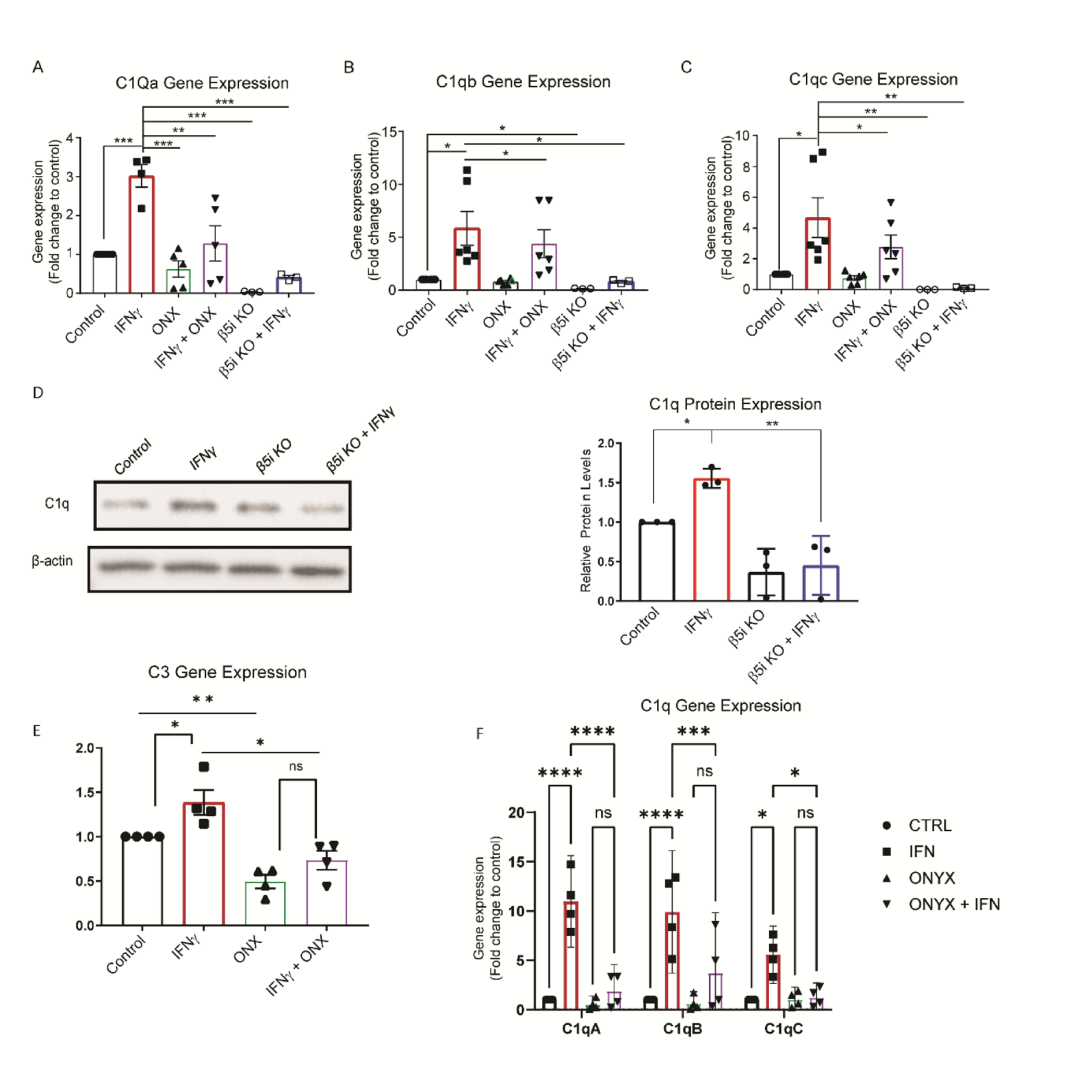
Immunoproteasome inhibition reduces IFNγ-dependent complement gene activation. Wild-type or β5i knockout BV-2 cells were treated with IFNγ in the absence and presence of immunoproteasome inhibitor, ONX-0914 and levels of C1q genes were analyzed. (**A**) Gene expression analysis of C1qa revealed a significant difference between treatment groups using one-way ANOVA ([F(5, 20) = 9.21], *p* < .001, *n* = 5). Tukey’s HSD post hoc test revealed that IFNγ resulted in a significant increase of C1qa compared to all other groups (control, *p* < .001; ONX-0914, *p* = .001; ONX + IFNg, *p* < .01; KO control, *p* < .001; K.O. IFNγ, *p* < .001). C1qa levels were not increased by IFNγ in b5i KO cells (*p* = .996, *n* = 3, t-test comparing two groups). (**B**) Gene expression analysis of C1qb revealed a significant difference between treatment groups using one-way ANOVA ([F(5, 20) = 10.56], *p* < .001, *n* = 5). Tukey’s HSD post hoc test revealed that IFNγ resulted in a significant increase of C1qb (control, *p* < .001; KO control, *p* < .001; KO IFNγ, *p* < .05). C1qb levels were not increased by IFNγ in b5i KO cells (*p* = .974, *n* = 3, t-test comparing two groups). (**C**) Gene expression analysis of C1qc revealed a significant difference between treatment groups using one-way ANOVA ([F(5, 24) = 10.56], *p* < .001, *n* = 6). Tukey’s HSD post hoc test revealed that IFNγ resulted in a significant increase of C1qc (control, *p* < .001; ONX + IFNγ, *p* = .012; KO control, *p* < .001; K.O. IFNγ, *p* < .01). C1qc levels were not increased by IFNγ in b5i KO cells (*p* > .999, *n* = 3, t-test comparing two groups) (**D**) Western blot analysis confirms that IFNγ treatment increases C1q protein levels in WT BV2 cells but not in β5i KO BV-2 cells. (**E**) Analysis of C3 gene expression revealed a significant difference between groups using one-way ANOVA (F(3,12) = 15.76, *p* < .001, *n* = 4). Tukey’s HSD post hoc test revealed that ONX-0914 treatment reduced C3 levels compared to control and IFNγ treatments (*p* < .01 and *p* ≤ .001, respectively). Further, ONX-0914 co-treatment with IFNγ reduced C3 levels compared to IFNγ alone (*p* = .002). (**F**) Gene expression analysis of C1qa, C1qb and C1qc in iPSC-derived microglia were determined by qRT-PRC (control *n* = 4, SMA *n* = 4; *p < .05, **p = .01, ***p < .001, ****P < .0001). Heat Map of Cytokine Levels. BV-2 cells were treated for 24 h then submitted to cytokine analysis. Values are mean pixel density of 4 independent experiments. * indicates a significant difference between control and IFNγ groups. ** (*p* < .05) indicates that ONX-0914 treatment reversed the IFNγ−dependent increase.

Reactive microglia have increased activation of the complement cascade, which can promote neural necrosis and inflammation^12^. BV-2 cells are an immortalized microglia cell line that, upon induction by IFNγ, express immunoproteasomes at approximately the same level as primary microglia^5,6^. In addition, BV-2 cells express complement genes similar to primary brain and retinal microglia^13^. Activation of the classical complement cascade begins when C1q, a fluid phase complement activator, binds to apoptotic cells or other microbes and debris^14^. Given that C1q promotes microglial reactivity, we sought to examine the role of the immunoproteasome on complement activation. We treated BV-2 cells with IFNγ and measured the C1q gene cluster, C1qa, C1qb and C1qc expression via qRT-PCR. We found that IFNγ treatment resulted in significantly higher gene expression of all 3 genes (*p <* .001, *p* < .001, and *p* < .001, respectively), which was abrogated in the presence of an immunoproteasome inhibitor (Fig. 1A-C; Supplemental Fig. 5). To confirm that loss of immunoproteasome activity suppressed complement gene activation we utilized BV2 cells where the catalytic β5i subunit was deleted (BV-2 β5i KO). Interestingly, we found low basal expression of each C1q gene in BV-2 β5i KO cells compared to wild type BV-2 cells (Fig. 1A-C). Moreover, IFNγ-dependent complement gene induction is blocked in BV-2 β5i KO cells (Fig. 1A-C), consistent with pharmacological inhibition of immunoproteasome activity. In addition to gene expression, C1q protein levels were upregulated by IFNγ in WT BV-2 cells. This IFNγ-dependent increase in C1q protein levels was reduced in β5i KO BV-2 cells compared to WT BV-2 cells (Fig. 1D).

C3 is a major component of the complement immune system, involved in both the classical and lectin complement pathways and is important for tagging pathogens, extracellular protein plaques such as amyloid beta, and other foreign complexes for phagocytosis^15,16^. C3 is synthesized under basal conditions but can be stimulated by IFNγ^17^. We measured C3 gene expression in the absence or presence of IFNγ and immunoproteasome inhibitor ONX-0914. We found that ONX-0914 treatment reduced basal levels of C3 expression compared to control (Fig. 1E). Further, IFNγ and ONX-0914 co-treatment reduced C3 expression compared to IFNγ alone (Fig. 1E). These data suggest that the immunoproteasome regulates C3 expression.

C3b tagged pathogens or complexes are recognized by microglia via the C3b receptor, Cr1 (also known as CD35)^18,19^. It has been previously reported that IFNγ decreases Cr1 levels on human monocytes in a dose dependent manner^20^. Given that immunoproteasome inhibition reduced the IFNγ-dependent increase in C1q, we sought to determine if IFNγ and/or the immunoproteasomes alter complement receptors in BV-2 microglia. Using flow cytometry and fluorescence-activated cell sorting (FACS) analysis, we found the levels of the complement receptors Cr1/2 or Cr3 levels were unchanged following IFNγ, ONX-0914 or co-treatment (Supplemental Fig. 1A-B). Interestingly, C1q receptor (CD93) expression was reduced in the presence of IFNγ, and this reduction was not significantly altered by ONX-0914 co-treatment (Supplemental Fig. 1C). The levels of the complement receptor C5aR (CD88) were also unchanged between groups (Supplemental Fig. 1D). Together, these data suggest that the immunoproteasome plays a critical role in complement activation by modulating C1q induction independent of complement receptor expression.

Given the growing evidence demonstrating differences in human and mouse microglia, we next sought to confirm that the immunoproteasome regulated IFNγ-dependent complement gene induction in human microglia cells. We utilized a doxycycline inducible human inducible pluripotent stem cells (iPSCs) microglia line that expresses crucial microglia markers and adopts a ramified morphology 8 days after differentiation. We then wanted to confirm that our iPSC-derived microglia expressed immunoproteasome subunits in the presence of IFNγ. We found all immunoproteasome catalytic subunits exhibited increased gene expression after exposure to IFNγ (Supplemental Fig. 2A-C). We treated iPSC-derived microglia with IFNγ and measured C1qa, C1qb and C1qc expression by qRT-PCR. Consistent with findings in mouse microglia, IFNγ treatment resulted in robust induction of C1q gene cluster, which was reversed in the presence of the immunoproteasome inhibitor (Fig. 1F). Together, our findings suggest that the immunoproteasome regulates IFNγ-dependent complement activation in mouse and human microglia.

### Phagocytosis is slowed by Immunoproteasome Inhibition

**Fig. 2:**
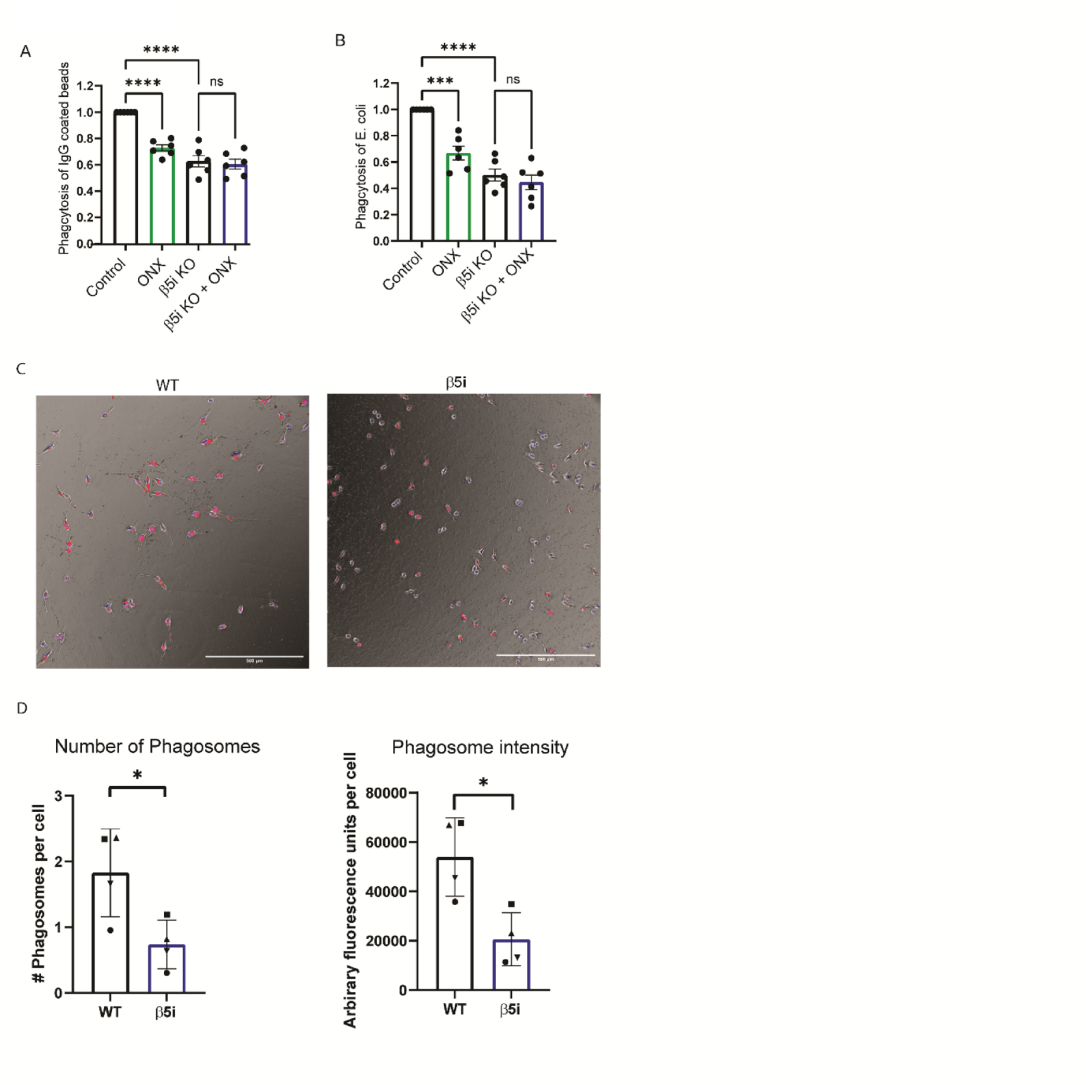
Phagocytosis is impaired by immunoproteasome inhibition. WT and β5i KO BV-2 cells were treated for 24 h with ONX-0914 prior to measuring phagocytosis by flow cytometry. To adjust for background, control cells that were not exposed to fluorescent beads were used for each experiment. (**A**) Phagocytosis of IgG-coated latex beads was significantly different between groups using one-way ANOVA ([F(3,10) = 7.68, *p* = .005). Tukey’s HSD post hoc test revealed that treatment with ONX-0914 resulted in significantly decreased phagocytosis compared to control (*p* = .032). (**B**) Phagocytosis of eGFP-expressing *E. coli* was measured after a 30-minute incubation by quantifying the percentage of cells that were GFP positive. Flow cytometry analysis revealed a significant difference between treatment groups using one-way ANOVA ([F(3,18) = 39.23], *p* < .001). Tukey’s HSD post hoc test revealed that treatment with ONX-0914 resulted in significantly decreased phagocytosis compared to control (*p* < .001).

Phagocytosis is a major component of innate immunity, and we have previously shown that immunoproteasome inhibition decreased phagocytosis of carboxylate-modified microspheres^6^. IgG-mediated phagocytosis is one mechanism employed by microglia to recognize and engulf pathogens^21^. To test whether immunoproteasomes were involved in IgG-mediated phagocytosis, we treated BV-2 cells with ONX-0914 for 24 h ours, then incubated cells with FITC-conjugated, IgG-coated latex beads. We found that ONX-0914 treatment reduced microglial-mediated phagocytosis of IgG-coated beads (Figure 2A). This finding was reproduced in β5i KO BV-2 cells, demonstrating that the effect on phagocytosis was not due to off -target effects of ONX-0914. We next tested if phagocytosis of E. coli, a more physiological relevant substrate, was impaired by inhibition of the immunoproteasome. We incubated cells with GFP-expressing E. coli for 30 m, in the presence or absence of ONX-0914, then subject the cells to flow cytometry to measure the percentage of cells that were GFP^+ 11^. BV-2 cells treated with ONX-0914 did not engulf E. coli as effectively as control cells, resulting in reduced percentage of GFP^+^ cells compared to control (Fig. 2B). Similar to the effect seen in phagocytosis of IgG-coated beads, the phagocytosis of E.coli was significantly reduced in the β5i KO BV-2 cells.

Additionally, WT and β5i KO BV-2 cells plated in glass chambers and treated with 2-micron beads coated with pHrodo-opsonized IgG showed a significant difference in phagocytosis at the end of 2 h. The β5i KO Bv-2 cells demonstrated a decrease in phagocytosis of these IgG coated beads, as seen through visualizing the red fluorescence of the internalized pHrodo label in cells when compared to WT Bv-2 cells (Fig. 2C). When comparing the fluorescence across cell types, WT BV-2 cells generate more phagocytosis of beads with higher fluorescence intensity than β5i KO Bv-2 cells (Fig. 2D). Taken together, these data indicate that the immunoproteasome has a global effect on phagocytosis, regardless of the substrate being engulfed, suggesting that it controls a cellular mechanism common to the different types of phagocytosis.

### IFNγ-dependent cytokines are modulated by Immunoproteasome Inhibition

**Fig. 3:**
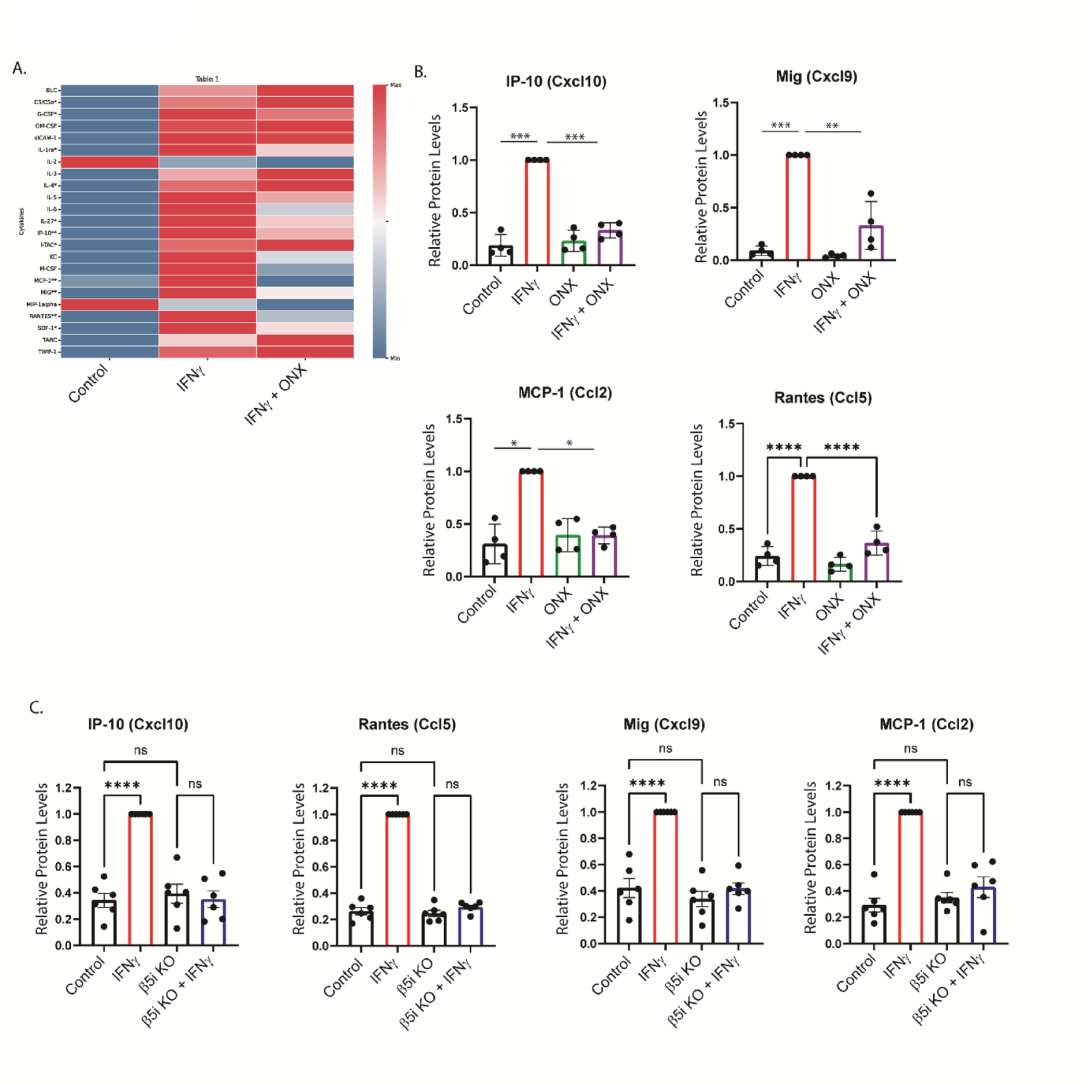
Immunoproteasomes mediate IFNγ-dependent cytokine production. BV-2 cells were treated for 24 h, and cytokine levels were measured using a Proteome Profiler assay. (**A**, **B**) The heat map displays the mean pixel density from four independent experiments (*n* = 4 per group). The color scale indicates the range of pixel density values from minimum (blue) to maximum (red). (*p* < .05) denotes a significant difference between the control and IFNγ treatment groups. ** (*p* < .05) indicates that co-treatment with ONX-0914 significantly reversed the IFNγ-dependent increase in cytokine levels. For a representative blot image and complete data, please see Supplemental Fig. 3 and the Supplemental Table, respectively. Statistical analysis using one-way ANOVA revealed that ONX-0914 treatment abrogated the IFNγ-dependent increase of Ip-10 ([F(3,11) = 104.4], *p* < .001). Tukey’s HSD post hoc analysis revealed that IFNγ increased Ip-10 levels compared to control (*p* < .001), ONX-0914 (*p* < .001), and ONX-0914 + IFNγ co-treatment (*p* < .001). In addition, there was a significant difference of Mig protein levels between treatment groups using one-way ANOVA ([F(3,11) = 18.61], *p* < .001). Tukey’s HSD post hoc analysis revealed that IFNγ treatment resulted in higher Mig protein levels than all other groups (Control *p* < .001; ONX-0914, *p* < .001; ONX-0914 + IFNγ, *p* = .003). MCP-1 levels were significantly different between groups using one-way ANOVA ([F(3,8) = 5.591], *p* = .02). Tukey’s HSD post hoc analysis revealed that IFNγ treatment increased MCP-1 levels compared to control (*p* = .035), which was reduced by ONX-0914 co-treatment (*p* = .029). Rantes protein levels were also different between treatment groups using one-way ANOVA ([F(3,12) = 24.18], *p* < .001). Tukey’s HSD post hoc analysis revealed that Rantes cytokine levels were significantly higher in the IFNγ treatment group compared to all other groups (Control, *p* < .001; ONX-0914, *p* < .001; ONX-0914, +IFNγ, *p* < .0001). (**C**) BV-2 β5i KO cells were treated with IFNγ for 24 h, and cytokine levels were measured using a Proteome Profiler assay. Ip-10, Mig, Rantes, and MCP-1 chemokine induction were abrogated in BV-2 β5i KO cells exposed to IFNγ, similar to ONX-0914 treatment.

In addition to complement activation and phagocytosis, cytokine release is a major feature of the innate immune response. During infection, release of cytokines and chemokines initiate the inflammatory response, facilitating debris clearance, immune cells recruitment and subsequent protection from infection. During trauma and ageing, however, blood brain barrier (BBB) dysfunction and recruitment of peripheral immune cells to the CNS are detrimental side-effects of cytokine production and release^22,23^. We next performed an unbiased cytokine protein array to examine the relative amounts of various cytokines produced by BV-2 cells exposed to IFNγ (Fig. 3A-B, Supplemental Fig. 3 and Supplemental Table). We found that IFNγ significantly increased the levels of 11 cytokines examined, compared to control (*n* = 4; *p* < .05). Furthermore, we found that co-treatment with ONX-0914 significantly reduced the levels of 4 of these cytokines compared to IFNγ treatment alone. Interestingly, not all the cytokines that were up-regulated by IFNγ were reduced by ONX-0914, suggesting that the immunoproteasome mediates a specific sub-set of IFNγ-induced cytokines. These results were consistent in β5i KO BV-2 cells, indicating that the observed effects on cytokine secretion are specifically related to immunoproteasome activity (Fig. 3C).

**Fig. 4:**
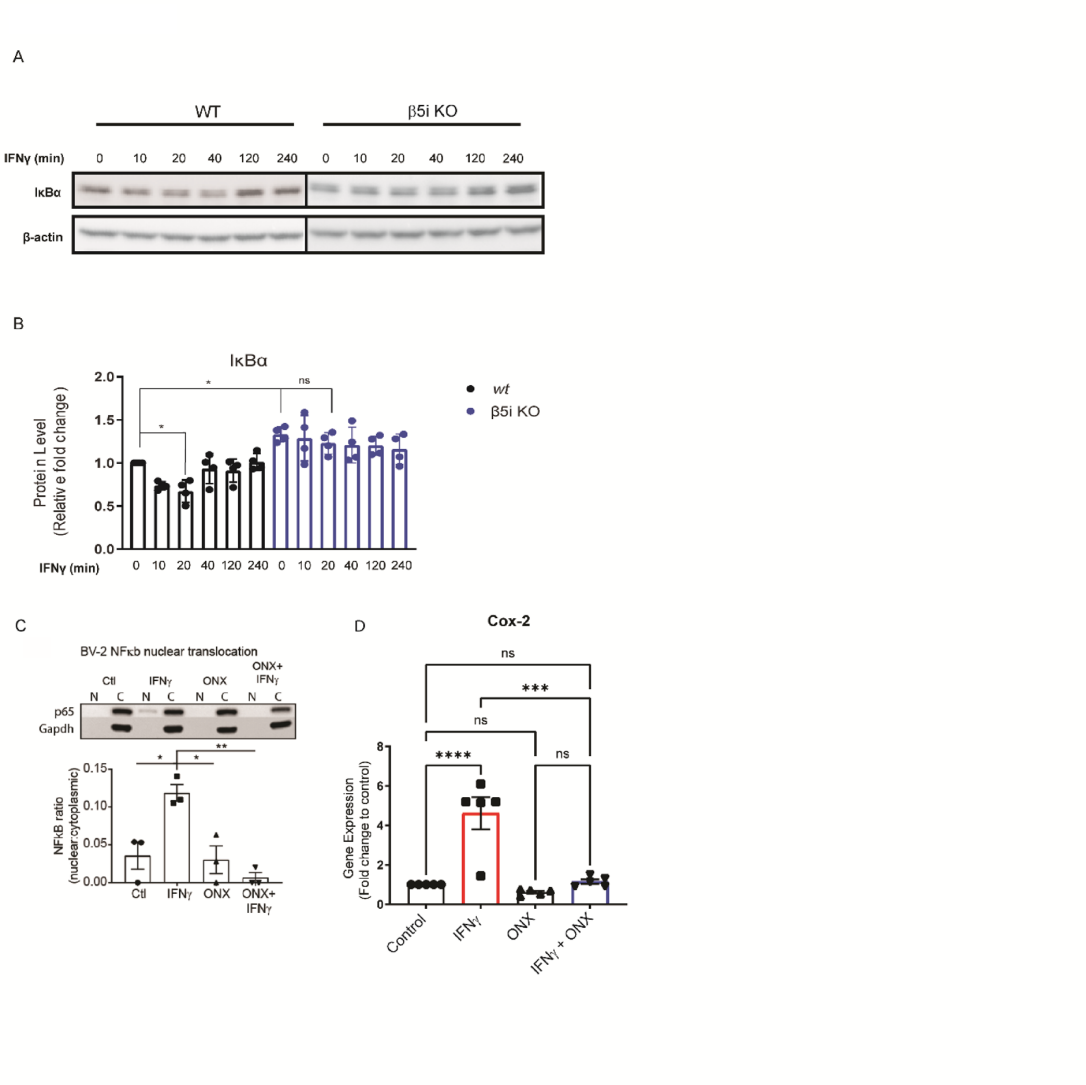
Altered degradation of IκBα in the absence of immunoproteasome activity. IκBα levels were measured in WT and β5i KO BV-2 cells in the absence and presence of IFNγ. (**A**) Representative Western blot of IκBα in WT BV-2 and β5i KO BV-2 βcells over a 240 min time course following IFNγ exposure. (**B**) Quantification of the data represented in (**A**). Statistical analysis using one-way ANOVA revealed a significant difference between groups ([F(3,28) = 12.75], *p* < .001). Tukey’s HSD post hoc analysis revealed that IFNγ treatment significantly reduced IkBa levels compared to control (*p* < .05) after 20 min. IkBa levels were unchanged in BV-2 b5i KO cells treated with IFNγ. *Individual comparisons indicated by asterisks likely used a t-test (n = 4, *p* < .05,* ns = no significance).* (**C**) NF-kB nuclear translocation was measured in BV-2 cells in the presence or absence of IFNγ and/or ONX-0914 by comparing the nuclear to cytoplasmic p65 ratios between groups. An ANOVA revealed a significant difference between groups using one-way ANOVA ([F(3,8) = 11.67], *p* = .002, *n* = 3). Tukey’s HSD post hoc analysis revealed that IFNγ treatment increased p65 nuclear to cytoplasmic ratio compared to control (*p* = .014). Co-treatment with IFNγ and ONX-0914 significantly decreased NF-kB activation compared to IFNγ treatment alone (*p* = .002), suggesting that immunoproteasome inhibition blocks the IFNγ-dependent activation of NF-kB. (**D**) Gene expression analysis of cox2 expression by qRT-PCR. Statistical significance between groups (*n* = 5) was determined using an independent samples t-test (***p < .001, ****p < .0001, ns = no significance).

The immunoproteasome mediated cytokines include Cxcl10, MIG, MCP-1, and RANTES. Cxcl10 (also known as IP-10) is a chemokine released by multiple cell types which binds to its receptor; Cxcr3, activating and facilitating microglia migration to sites of injury^24^. Binding of Cxcl10 to neurons has been linked to neuronal death, highlighting the importance of understanding the mechanisms of modulating Cxcl10 levels^25^. We found that Cxcl10 is increased following IFNγ treatment, however when cells are co-treated with ONX-0914, this effect is diminished (Figs. 3A-B and 4). Another ligand that binds to Cxcr3, resulting in microglia activation and immune cell recruitment, is MIG (also known as Cxcl9)^26^. Here we found that MIG levels are increased following IFNγ treatment, which is abrogated in the presence of ONX-0914.

Monocyte Chemotactic and Activating Factor, MCP-1, (also known as Ccl2) is released by microglia during trauma and infection, and is also a key modulator of neuropathic pain^27^. Microglia lacking MCP-1 are less activated and have reduced motility, resulting in improved histological and behavioral outcomes following intracerebral hemorrhage in mice^28^. We found that IFNγ increased MCP-1 in BV-2 microglia, an effect that was blocked when cells were co-treated with the immunoproteasome inhibitor (Fig. 3A-B).

RANTES (also known as Ccl5) is a chemokine that is up-regulated in response to trauma and other neurological diseases that, in addition to its chemo attractant properties, induces microglia to a pro-inflammatory state^29^. Inhibition of RANTES reduces neuroinflammation and decreases BBB permeability, thus decreasing RANTES levels during chronic inflammation could prove useful^30^. Here we show that IFNγ significantly increases RANTES protein levels, but not when cells were co-treated with ONX-0914 (Fig. 3A-B). In addition, we showed that the IFNγ-dependent induction of each chemokine was blocked in BV-2 cells where the β5iβ subunit was knocked out, confirming the findings observed with ONX-0914 treatment (Fig. 3C).

Given human and mouse chemokines may be differentially regulated, we next sought to determine if the chemokines we identified in mouse BV-2 cells were similarly immunoproteasome-dependent in human microglia. Inducible human iPSC-derived microglia were exposed to IFNγ and the expression of the select chemokines were determined by qRT-PCR. We found that Cxcl10, MCP-1, and RANTES expression were increased in the presence of IFNγ (Supplemental Fig. 4 and Supplemental Fig. 7). This IFNγ-dependent induction of each chemokine was suppressed when cells were co-treated with ONX-0914. We did not detect the presence of MIG in iPSC-derived microglia. Taken together, we show that the immunoproteasome mediates multiple chemokines that are primarily related to chemotaxis, responsible for microglial motility, and immune cell recruitment.

### Altered microglia innate immune response is due to Immunoproteasome regulation of NF-κB

In addition to complement activation and phagocytosis, MIG^31^, RANTES^32^, MCP-1^33^ and Ip-10^34^ have all been shown to be transcriptionally regulated by NF-κB, so we sought to determine whether loss of the immunoproteasome subunit β5i altered IκBα degradation, a surrogate marker of NFκB activation. We treated WT and β5i KO BV-2 cells with IFNγ over the course 4 h and analyzed IκBα protein levels. We found that IκBα was significantly reduced after 20 min of IFNγ treatment in WT BV-2 cells (Fig. 4A-B). However, later time points resulted in no significant change compared to untreated cells (0 min) (Fig. 4A-B). This suggests that IFNγ signaling exhibits peak IκBα degradation 20 min post-stimulation and begins resynthesis at 40 min post-stimulation. Interestingly, IκBα was stabilized in the β5i KO BV-2 cells as we observed no significant change in IκBα degradation over time compared to control (Fig. 4A, B). Concomitantly, IκBα basal levels were significantly greater in β5i KO BV-2 cells compared to WT BV-2 cells prior to IFNγ treatment (Fig. 4A, B; Supplemental Fig. 6).

To assess the impact of immunoproteasome inhibition on NF-κB signaling, we examined the nuclear translocation of the p65 subunit. Microglia were treated with IFNγ to activate NF-κB, with or without ONX-0914. Following treatment, nuclear and cytoplasmic fractions were collected. Western blot analysis was performed using antibodies against p65 and GAPDH serving as loading controls for the cytoplasmic fractions. IFNγ treatment increased p65 in the nuclear fraction, indicating NF-κB activation, which was blocked by ONX-0914 (Fig. 4C).

Cox-2 is a well-known downstream gene target of NF-κB which, when NF-κB is inhibited, Cox-2 gene expression is also inhibited. As a measure of NF-κB activity, we measured gene expression levels of Cox2 in the absence and presence of IFNγ and/or ONX-0914 treatment. We found that IFNγ treatment increased Cox2 levels (Fig. 4D). The IFNγ-dependent increase in Cox-2 expression was blocked when cells were co-treated with ONX-0914 (Fig. 4D). These data are consistent with the reduced IκBα degradation in β5i KO BV-2 cells, providing further evidence that immunoproteasome inhibition decreases IFNγ-dependent NF-κB activation.

### NADH reduces Immunoproteasome levels and complement gene expression

**Fig. 5:**
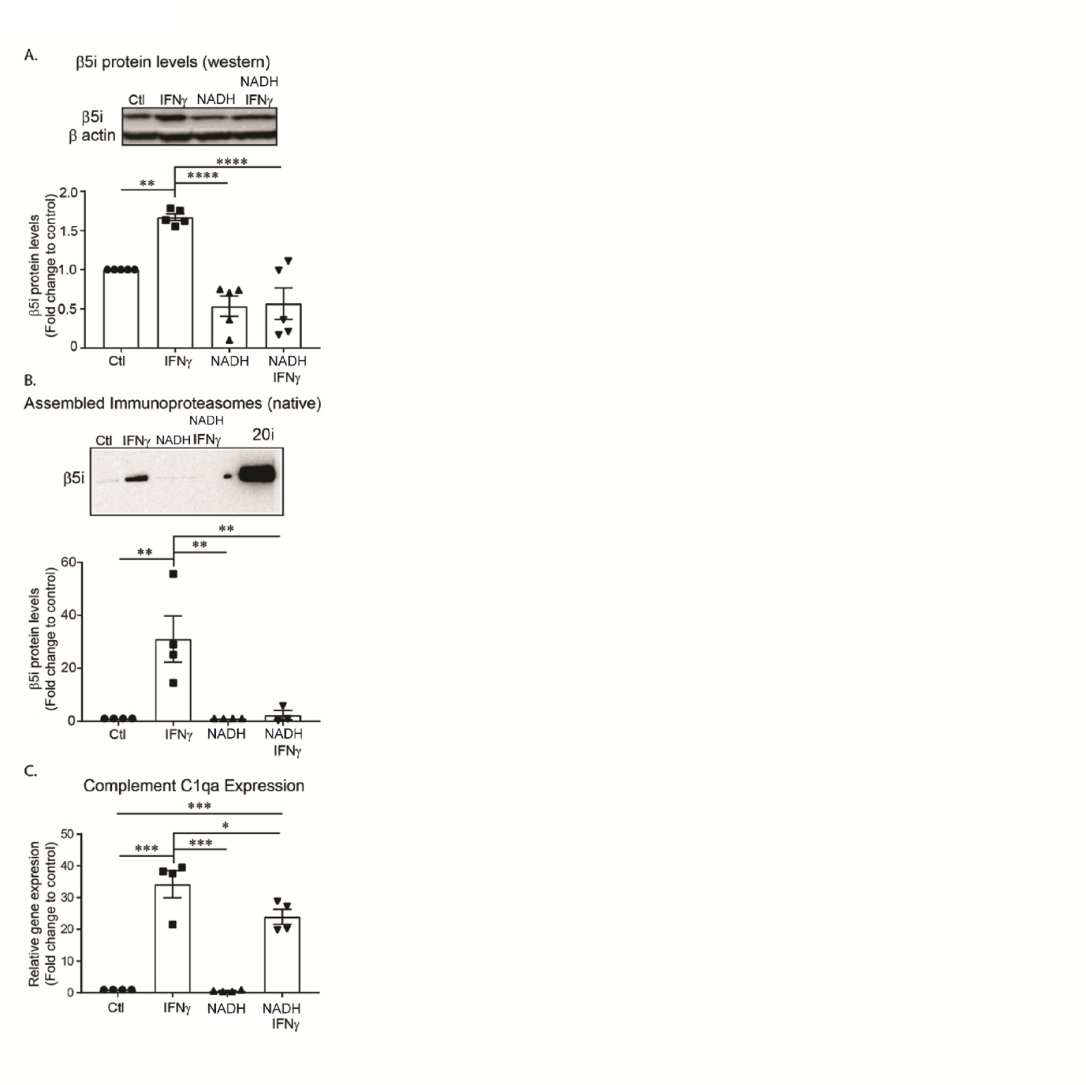
NADH blocks formation of the immunoproteasome. BV-2 cells were pre-treated with NADH for 24 h, then treated with IFNγ for an additional 24 h. Relative amounts of immunoproteasome protein levels were quantified. (**A**) Western blot analysis revealed a significant difference between treatment groups using one-way ANOVA ([F(3, 16) = 19.04], *p* < .001, *n* = 4). Tukey’s HSD post hoc analysis revealed that IFNγ increased total β5i protein levels compared to all groups (Control, *p* = .006; NAD, *p* < .001; NADH + IFNg, *p* < .0001). Further, IFNγ did not significantly increase β5i protein levels in cells pre-treated with NADH (*p* = .997, t-test comparing two groups). (**B**) Assembled immunoproteasomes (20i represents purified positive control) were measured using native gel electrophoresis. Analysis revealed that there was a significant difference between treatment groups using one-way ANOVA ([F(3, 11) = 9.845], *p* = .002, *n* = 4). Tukey’s HSD post hoc analysis revealed that IFNγ increased the amount of assembled immunoproteasomes compared to all treatment groups (Control, *p* = .003; NADH, *p* = .004; NAD + IFNγ, *p* = .009). Interestingly, when cells are pre-treated with NADH, immunoproteasomes are not increased in response to IFNγ (*p* = .996, t-test comparing two groups). (**C**) To determine if NADH treatment would successfully reduce complement activation in BV-2 cells, we pre-treated with NADH, then measured gene expression of complement activator C1qa. An ANOVA revealed a significant treatment effect using one-way ANOVA ([F(3,12) = 48.22, *p* < .001). Tukey’s HSD post hoc analysis revealed a significant increase of C1qa gene expression in response to IFNγ treatment (*p* < .001), an effect that was reduced by NADH pre-treatment (*p* = .049).

Extracellular Nicotinamide Adenine Dinucleotide (NAD) concentrations are basally low, however, it is released in high amounts during inflammation^35^. Exogenous NAD has been previously shown to decrease microglia activation following traumatic brain injury^36^. Interestingly, it has been reported that endogenous NAD^+^ can get taken up by cells, converted to NADH, and stabilize the constitutive proteasome^37^. Thus, we looked to determine if NADH treatment modulated the microglia inflammatory response via stabilization of the constitutive proteasome and subsequent suppression of immunoproteasome assembly. Pre-treatment of cells with NADH blocked IFNγ-dependent induction of the β5i subunit (Fig. 5A) and assembly of the immunoproteasome (Fig. 5B). We found a significant reduction in the expression of the complement protein C1qa, demonstrating a functional consequence of NADH treatment (Fig. 5C). These data suggest that assembly of the immunoproteasome can be blocked by stabilizing the constitutive proteasome.

## Discussion

Our group and others have shown previously that he proteasome is responsible for the selective degradation of most intracellular proteins and regulates inflammatory and immune responses^6,8^. Based on transcriptome analysis from our previous study we hypothesized that the immunoproteasome played a role in both adaptive and innate immune responses. The goal of this study was to examine which aspects of the microglial innate immune response are mediated by the immunoproteasome. We found three central components of the innate immune response, activation of the complement cascade, phagocytosis and cytokine release, were all impacted by immunoproteasome inhibition, consistent with a critical role of the immunoproteasome in the microglial innate immune response.

Microglia are the dominant source of complement activation protein, C1q, in the brain^38^. Activation of the complement cascade in microglia has implications seen in multiple neurodegenerative diseases. Microglial reactivity, complement activation and pro-inflammatory cytokine release are commonly reported in Alzheimer’s Disease (AD) (For review^39^). However, recent evidence shows microglia may not just respond to injury, but may actually precede and exacerbate neuropathology^40^. While our study highlights the role of the immunoproteasome in complement activation, other regulatory mechanisms warrant further investigation. For example, previous research has shown that IFNγ can decrease Cr1 levels on human monocytes in a dose-dependent manner. However, it is important to note that this specific effect has not yet been directly demonstrated in human microglia, and further research is needed to confirm whether this regulatory mechanism is also relevant in these cells. Understanding the complex interplay between IFNγ, complement receptors, and the immunoproteasome in microglia may help to identify new therapeutic targets for neuroinflammatory conditions.

One of the neuropathological hallmarks of AD is synaptic loss, which microglia have been shown to facilitate via complement mediated synaptic pruning^41,42^. In neurodegenerative diseases such as AD and Parkinson’s disease (PD), as well as after traumatic brain injury (TBI), phagocytosis is critical for clearance of dying cells. Contrary to this, several studies have suggested that phagocytosis may exacerbate neuronal death during inflammation, promoting neurodegeneration^43–47^. Bacterial infection is one of the major activators of the innate immune system and there is some evidence suggesting a role of bacterial infection in the pathogenesis of AD ^48^. Thus, tight regulation of phagocytosis during and after inflammation is critical to maintain a healthy balance between debris clearance and unwanted neuronal phagocytosis. In the current study, we demonstrate that genetic ablation of β5i and -treatment with immunoproteasome inhibitors slows phagocytosis through different mechanisms, suggesting that there are potentially multiple converging immunoproteasome-dependent phagocytosis pathways in microglia. Better understanding of this process will allow us to devise mechanisms to maintain the proper balance between clearance of debris and over-activation of phagocytosis.

In addition to phagocytic activity, microglia rapidly release several cytokines and chemokines in response to infection or injury which act to limit the toxic insult and recruit other immune cells. In the current work we found that immunoproteasome inhibition selectively alters levels of some cytokines, but does not have a global effect on all cytokines measured. Cxcl10, RANTES, MIG, and MCP-1 are all chemoattractant molecules that help facilitate cellular migration, recruitment of microglia and peripheral immune cells to the site of damage. Following a CNS lesion, microglia migrate to the site of damage in an IP-10 dependent manner, resulting in dendritic degeneration. IP-10 receptor knockout, spares dendritic degeneration by microglia^24^. Our finding reveals that IFNγ stimulates microglia to release specific chemoattractants through an immunoproteasome-dependent mechanism, potentially influencing neuronal degeneration following an injury. It is important to consider that the immunoproteasome-mediated effects in microglia may extend beyond their intracellular functions to influence neighboring cells, such as neurons. For instance, CXCL10, a chemokine released by microglia, can bind to neurons and induce neuronal death. Additionally, CXCL9, another chemokine identified in our study, has been implicated in neuronal hyperexcitability following injury. Prostaglandin-endoperoxide synthase 2 (COX2), an enzyme involved in inflammation, is also known to be regulated by the immunoproteasome and can contribute to neuronal damage and neuroinflammation. These findings highlight the potential impact of microglial immunoproteasome activity on neuronal function and survival, emphasizing the complex interplay between these two cell types in the neuroimmune environment.

Upon degradation of IκBα, the p65 subunit of NFκB is translocated to the nucleus to activate the transcription of a large amount of immune response-related genes. There have been conflicting reports regarding whether the immunoproteasome more efficiently degrades IκBα compared to the constitutive proteasome^49,50^. Here we report that in response to IFNγ, IκBα is stabilized in β5i KO BV-2 cells, suggesting that the immunoproteasome is essential for efficient IκBα degradation. This finding implies that the immunoproteasome regulates IκBα turnover and subsequent IFNγ-dependent NFκB activation and reveals that the immunoproteasome plays a crucial role in regulating IκBα degradation, thereby influencing IFNγ-dependent NFκB activation. This observation aligns with previous research highlighting the intricate relationship between the immunoproteasome and NFκB signaling. While the precise mechanisms underlying this interaction remain an area of ongoing investigation, our findings suggest that the immunoproteasome may be essential for efficient IκBα degradation in response to IFNγ stimulation. This process could have broader implications for the inflammatory response, as NFκB regulates the expression of various inflammatory mediators, including COX2. Further research is needed to fully elucidate the interplay between IFNγ, the immunoproteasome, NFκB, and COX2 in microglia and their collective impact on neuroinflammation. NAD is a coenzyme that is found in all cells and is most commonly studied for its role in metabolism. However, recent studies have implicated decreased NAD levels in aging and neurodegeneration^51^. Another, somewhat less investigated function of the reduced form of NAD, NADH, is its ability to bind and stabilize the constitutive proteasome^37^. We and others have reported that IFNγ results in the loss of constitutive proteasome levels; however, we recently suggested that immunoproteasome subunits are inserted into pre-existing constitutive proteasomes, following disassembly^6,52^. Interestingly, data from this study support our previous findings; as cells pre-treated with NADH, which stabilizes the constitutive proteasome, have reduced levels of immunoproteasomes compared to IFNγ treatment alone.

In summary, we provide evidence that the immunoproteasome is critically involved in the major aspects of innate immunity, including the complement system, phagocytosis and cytokine release. In addition, the data presented here suggest that immunoproteasomes modulate microglia immune response by regulating NF-κB activation. Finally, we show that NADH, a known proteasome interacting co-factor, can reduce levels of assembled immunoproteasomes in response to IFNγ and, by doing so, reduce the immune response.

## Supplementary Information

Below is the link to the electronic supplementary material.

Supplementary Material 1

Supplementary Material 2

Supplementary Material 3

Supplementary Material 4

Supplementary Material 5

Supplementary Material 6

Supplementary Material 7

Supplementary Material 8

Supplementary Material 9

## Data Availability

The data that support the findings of this study are available from the corresponding author upon reasonable request.
