# Supplementary material for: Key features of the innate immune response is mediated by the immunoproteasome in microglia
Source: Sci Rep. 2025 Nov 21;15:41349. doi: 10.1038/s41598-025-25341-5 (PMC12638985; doi:10.1038/s41598-025-25341-5)
Supplement: Supplementary file 1 — Supplementary Material 1 [file 41598_2025_25341_MOESM1_ESM.docx]

**Supplemental Figure 1. Complement receptors expression are not altered by ONX**

**treatment.**

Complement receptors were measured in BV-2 cells by flow cytometry A. Complement receptor

(CR) 1/2 was unchanged between groups ([F(3,12)=1.08], *p*=.391). B. CR3 was also unchanged

between groups ([F(3,16)=.317], *p*=.812). C. Surface levels of CD93 was significantly different between groups ([F(3,8)=11.49], *p*=.002). *Post hoc* analysis revealed that IFNγ decreased levels of CD93 compared to control (*p*=.005) and ONX (*p*=.028) treatments. In addition, ONX co-treatment did not reverse the IFNγ-dependent decrease compared to control, thus ONX co-treatment was significantly lower than control (*p*=.008). D. There was a main effect of treatment on CD88 levels, ([F(3,8)=4.10], *p*=.048), however *post hoc* analysis revealed that there were no differences between groups.

**Supplemental Figure 2. IFNγ induces the immunoproteasome in human iPSC derived microglia** Gene expression for **A**. PSMB8 (β5i), **B**. PSMB9(β1i), **C**. PSMB10 (β2i) analyzed in iPSC-derived microglia 24 hours after exposure to IFNγ relative to vehicle control (n=3) *p<0.05 ****p<0.0001. All groups were analyzed by unpaired t test. Bars show mean ±SEM.

**Supplemental Figure 3. Immunoproteasomes mediate IFNγ-dependent cytokine**

**production.** BV-2 microglial cells were treated for 24 hours as indicated. Cytokine levels in

extracted cell lysates were then measured using a Proteome Profiler Human Cytokine Array.

Representative blot images are shown.

**Supplemental Figure 4.** **Expression of Cxcl10 and Ccl2 in IFNγ-treated β5i KO BV-2 cells and human iPSC-derived microglia. A.** Cxcl10 and Ccl2 protein levels are upregulated by IFNγ. Western blot analysis confirms that Cxcl10 and Ccl2 are induced in the presence of IFNγ. Induction of both proteins is blocked in BV-2 cells lacking the β5i subunit. **B.** Gene expression for Ccl5, Cxcl10 and Ccl2 analyzed in iPSC-derived microglia 24 hours after exposure to IFNγ in the present or absence of ONX compared to vehicle control (n=4) ****p<0.0001. All groups were analyzed by unpaired t test. Bars show mean ±SEM.

**Supplemental Figure 5. Full, uncropped Western blots.** This figure provides the complete, uncropped Western blot images corresponding to the cropped blots presented in the main figure 1 of the manuscript.

**Supplemental Figure 6. Full, uncropped Western blots.** This figure provides the complete, uncropped Western blot images corresponding to the cropped blots presented in the main figure 4 of the manuscript.

**Supplemental Figure 5. Full, uncropped Western blots.** This figure provides the complete, uncropped Western blot images corresponding to the cropped blots presented in the main Supplemental figure 4 of the manuscript.
