# Supplementary material for: Key features of the innate immune response is mediated by the immunoproteasome in microglia
Source: Sci Rep. 2025 Nov 21;15:41349. doi: 10.1038/s41598-025-25341-5 (PMC12638985; doi:10.1038/s41598-025-25341-5)
Supplement: Supplementary file 2 — Supplementary Material 2 [file 41598_2025_25341_MOESM2_ESM.docx]

|  | **Control** | **IFNγ** | **ONX-0914** | **IFNγ+ONX-0914** |
| --- | --- | --- | --- | --- |
| BLC | 866.50 | 1218.27 | 1508.71 | 1337.00 |
| C5/C5a* | 467.32 | 811.13 | 969.00 | 891.92 |
| G-CSF* | 151.79 | 465.61 | 534.88 | 411.57 |
| GM-CSF | Undetected | 164.42 | 275.10 | 174.34 |
| sICAM-1 | 14824.16 | 22037.30 | 21240.40 | 21870.84 |
| IL-1ra* | 3096.32 | 10518.27 | 4585.73 | 7466.45 |
| IL-2 | 808.91 | 373.07 | 117.85 | 264.98 |
| IL-3 | 792.38 | 1260.42 | 1407.41 | 1469.89 |
| IL-4* | 416.19 | 767.21 | 922.63 | 825.54 |
| IL-5 | 84.65 | 380.33 | 393.02 | 290.82 |
| IL-6 | Undetected | 168.73 | 209.51 | 60.62 |
| IL-27* | 51.76 | 387.53 | 189.89 | 255.20 |
| IP-10** | 1389.76 | 21515.35 | 1971.25 | 14879.69 |
| I-TAC* | 429.45 | 752.20 | 907.04 | 800.80 |
| KC | 93.74 | 379.99 | 384.28 | 208.07 |
| M-CSF | 9.02 | 401.14 | 453.96 | 74.95 |
| MCP-1** | 1029.59 | 2077.57 | 1458.85 | 885.17 |
| MIG** | Undetected | 3707.30 | 35.24 | 1916.18 |
| MIP-1alpha | 7084.88 | 4003.13 | 9256.91 | 2516.37 |
| RANTES** | Undetected | 932.45 | 47.91 | 270.62 |
| SDF-1* | 414.34 | 1229.00 | 1130.33 | 866.24 |
| TARC | 834.81 | 1133.93 | 1417.26 | 1343.51 |
| TIMP-1 | 726.10 | 1129.15 | 1389.80 | 1177.04 |

**Supplemental Table: Mean cytokine levels.** BV-2 cells were treated for 24 hours then submitted to cytokine analysis. Values are mean pixel density of 4 independent experiments. * indicates a significant difference between control and IFNγ groups. ** (p<0.05) indicates that ONX-0914 treatment reversed the IFNγ−dependent increase.
