# Supplementary figures and images for: Key features of the innate immune response is mediated by the immunoproteasome in microglia
Source: Sci Rep. 2025 Nov 21;15:41349. doi: 10.1038/s41598-025-25341-5 (PMC12638985; doi:10.1038/s41598-025-25341-5)

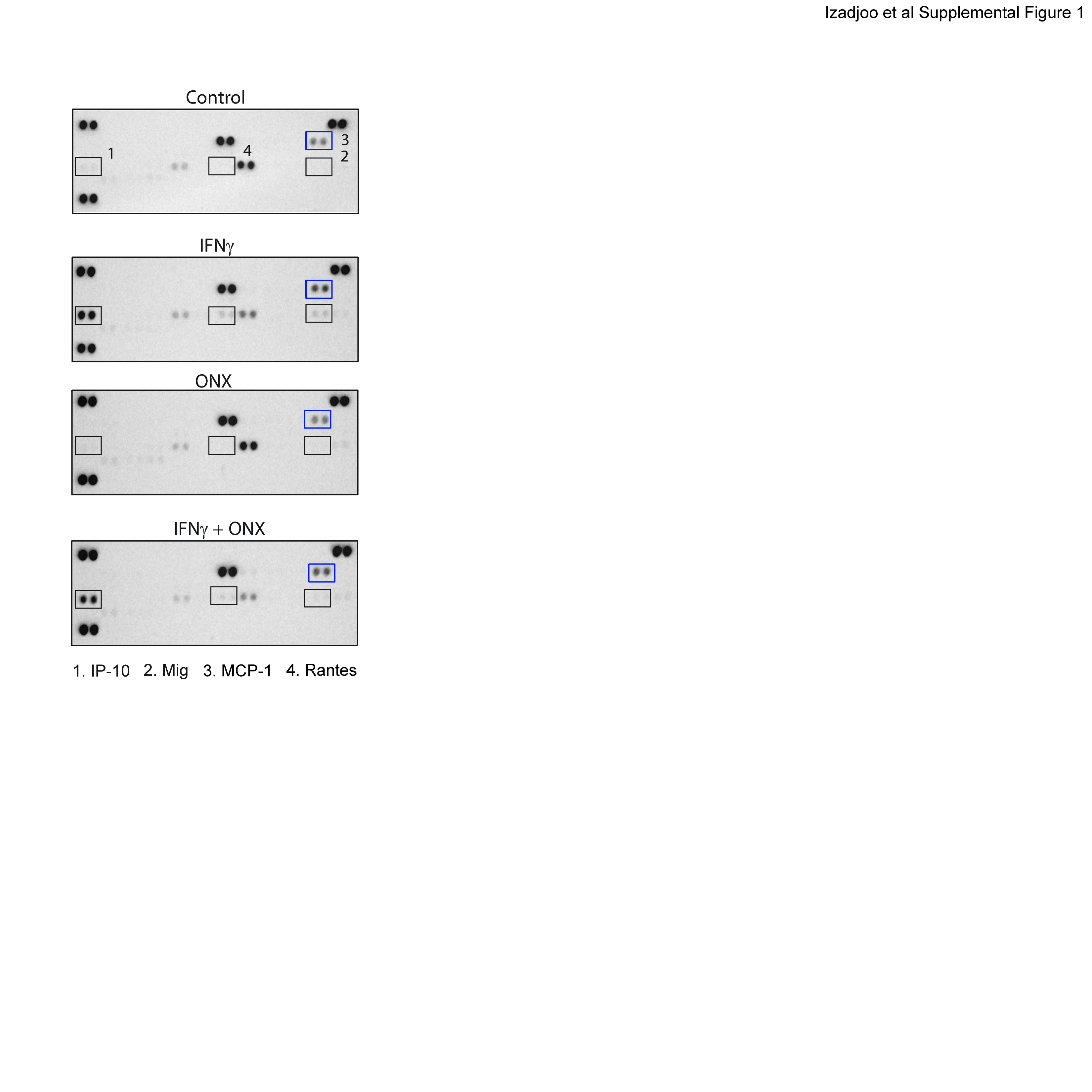

Supplement: Supplementary file 3 — Supplementary Material 3 [file 41598_2025_25341_MOESM3_ESM.tif]

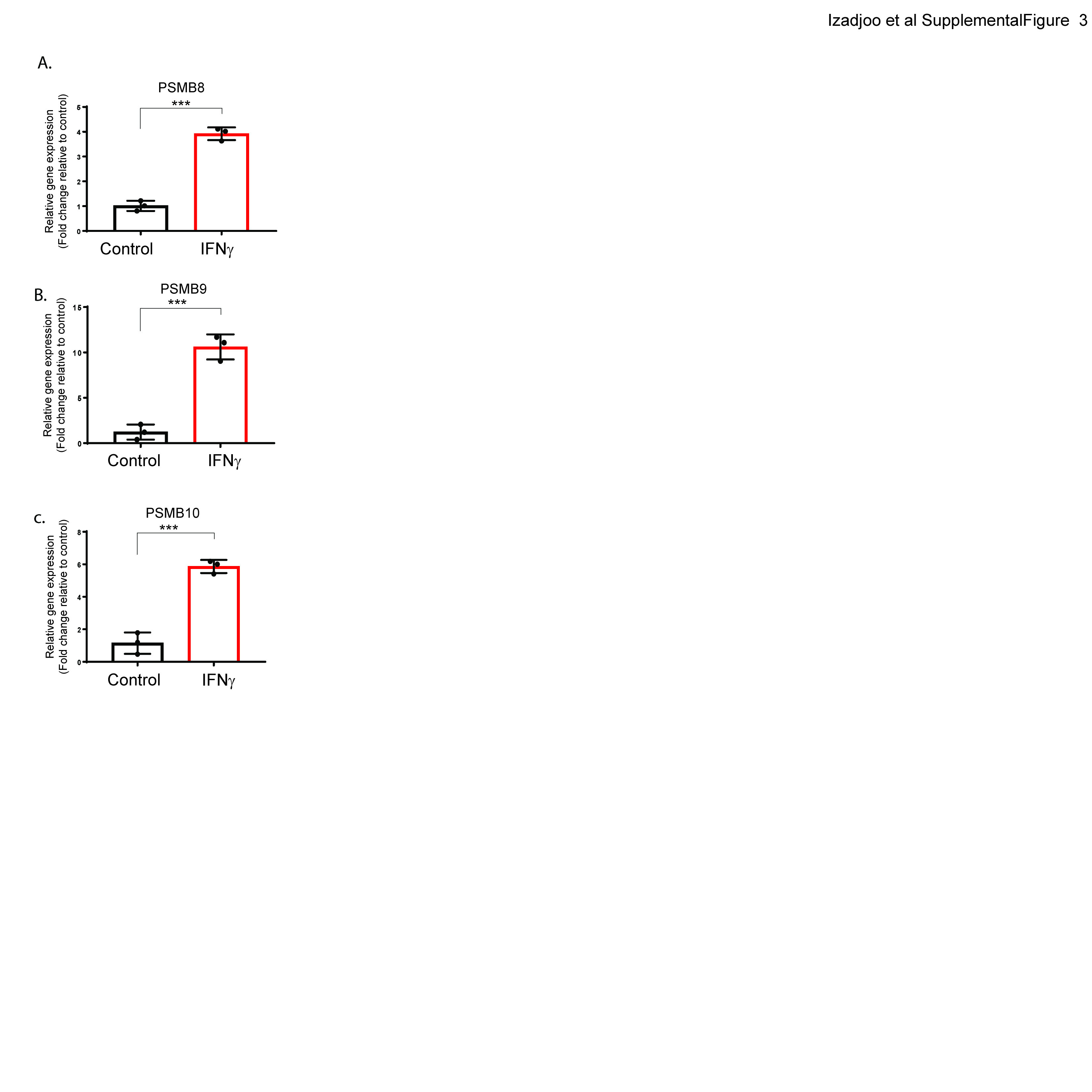

Supplement: Supplementary file 4 — Supplementary Material 4 [file 41598_2025_25341_MOESM4_ESM.tif]

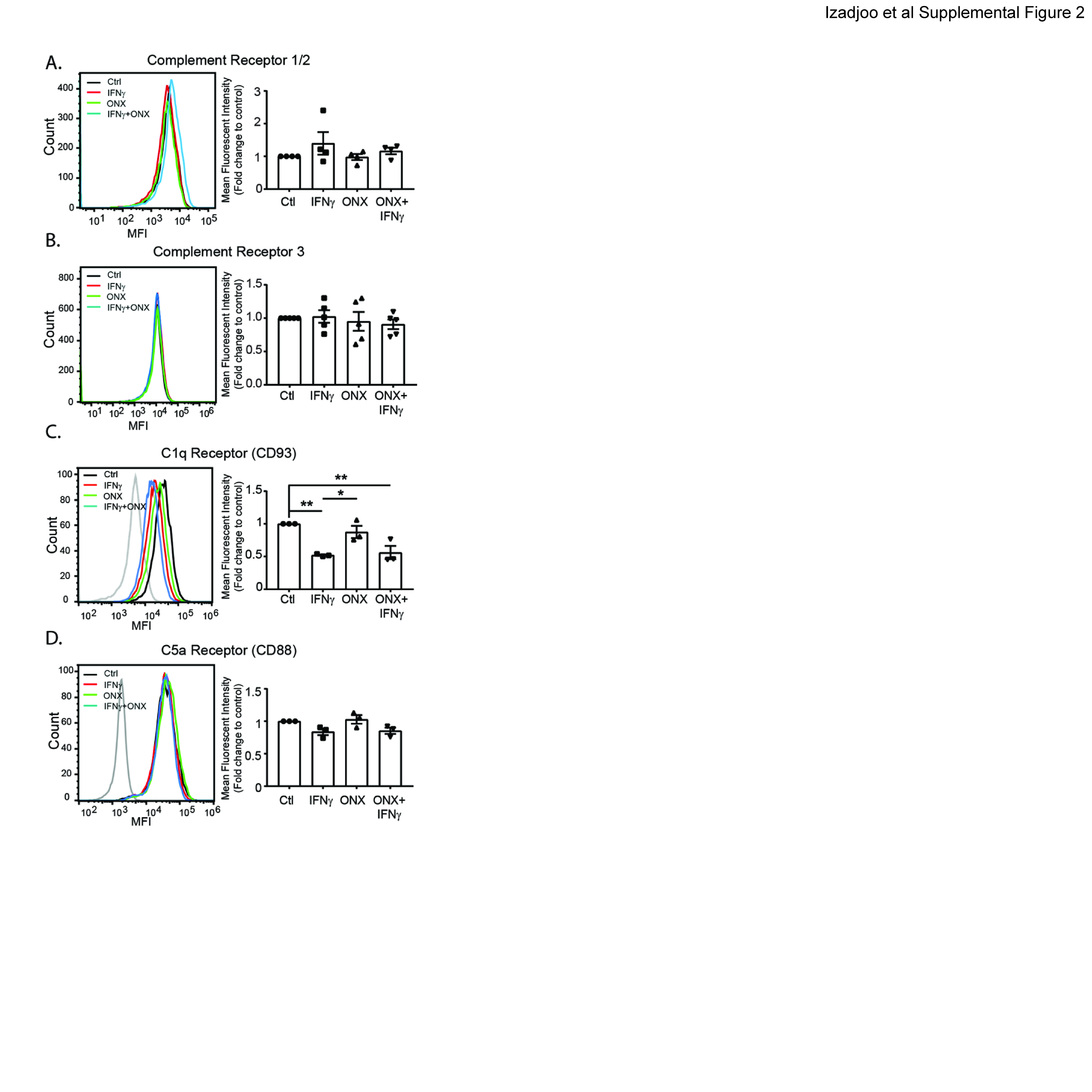

Supplement: Supplementary file 5 — Supplementary Material 5 [file 41598_2025_25341_MOESM5_ESM.tif]

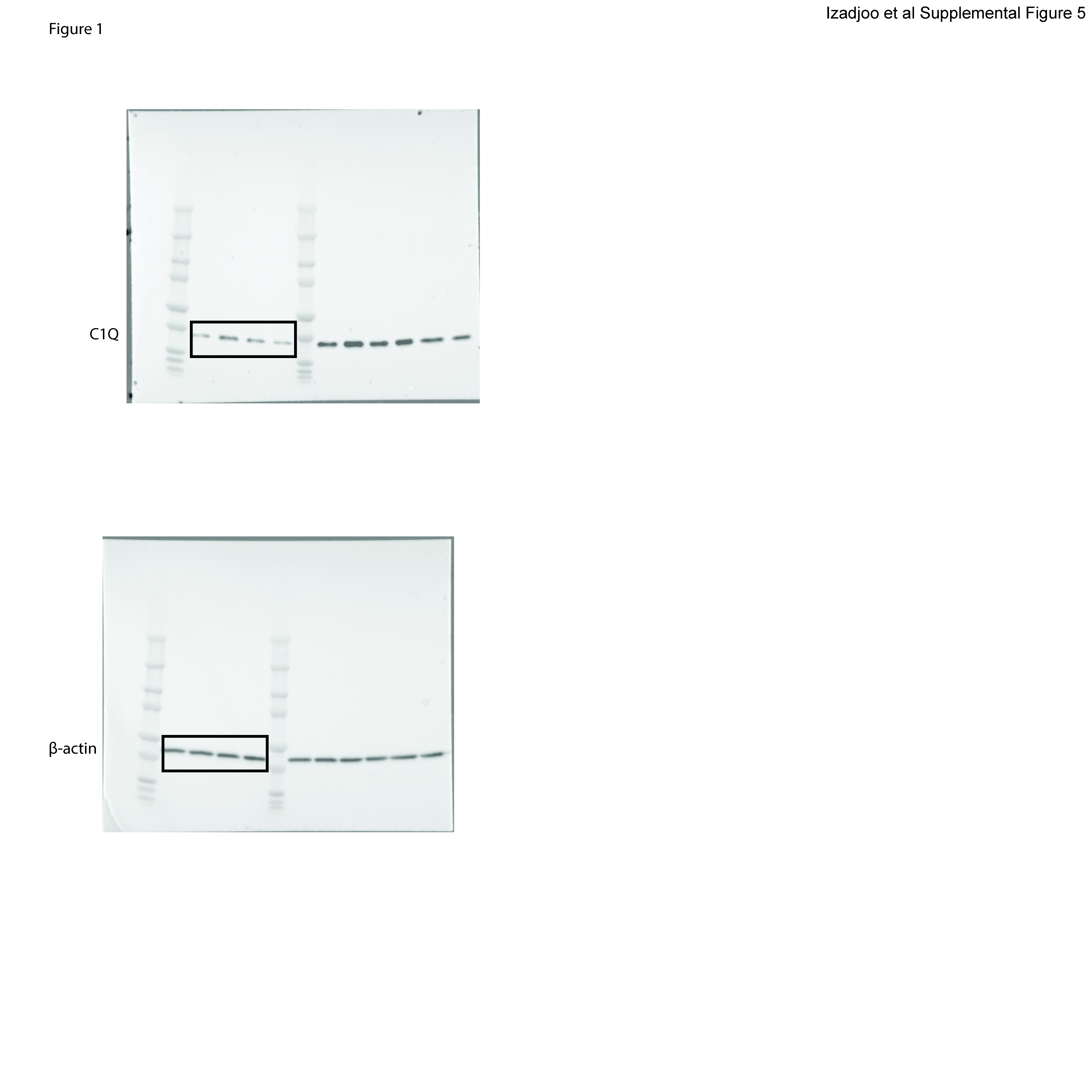

Supplement: Supplementary file 7 — Supplementary Material 7 [file 41598_2025_25341_MOESM7_ESM.tif]

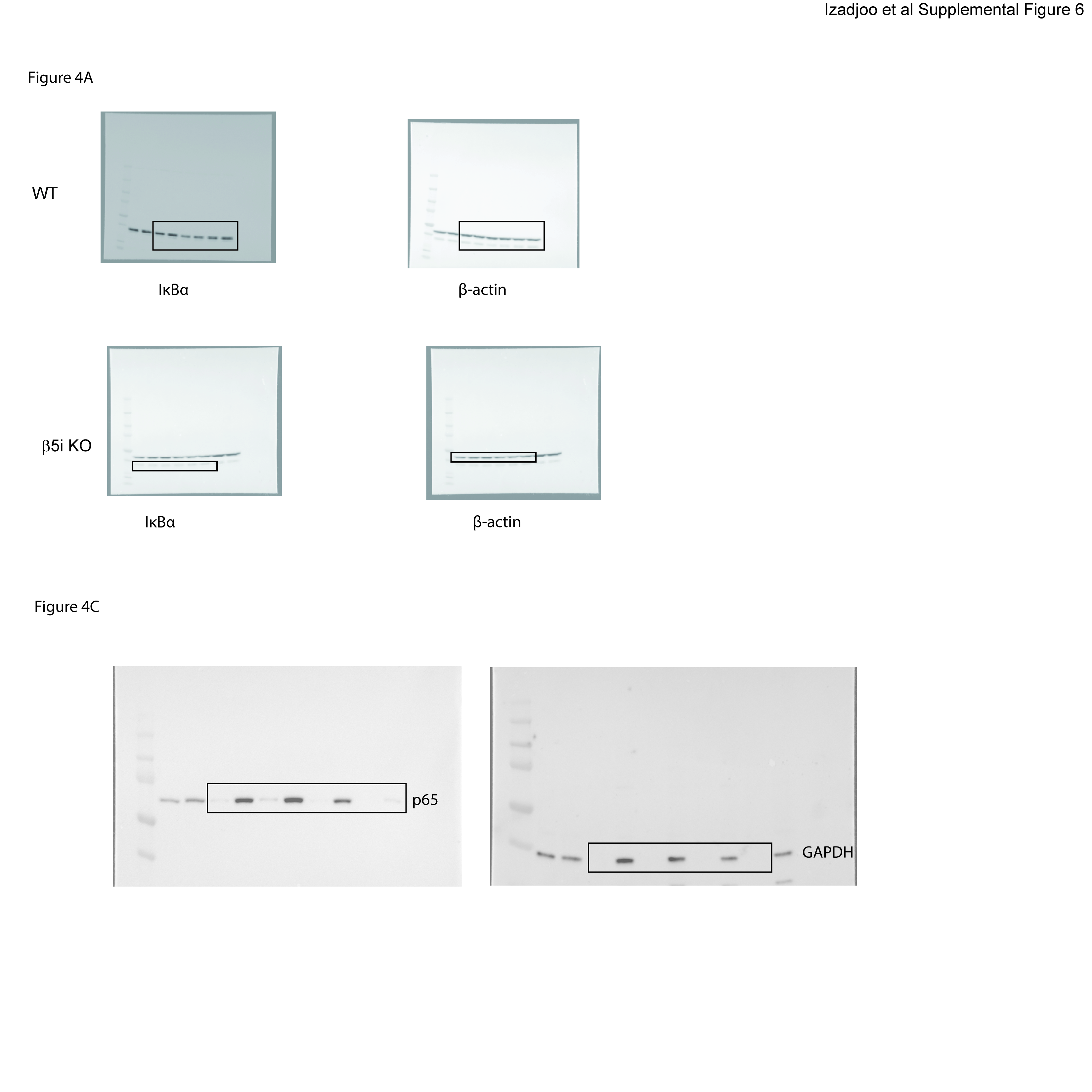

Supplement: Supplementary file 8 — Supplementary Material 8 [file 41598_2025_25341_MOESM8_ESM.tif]

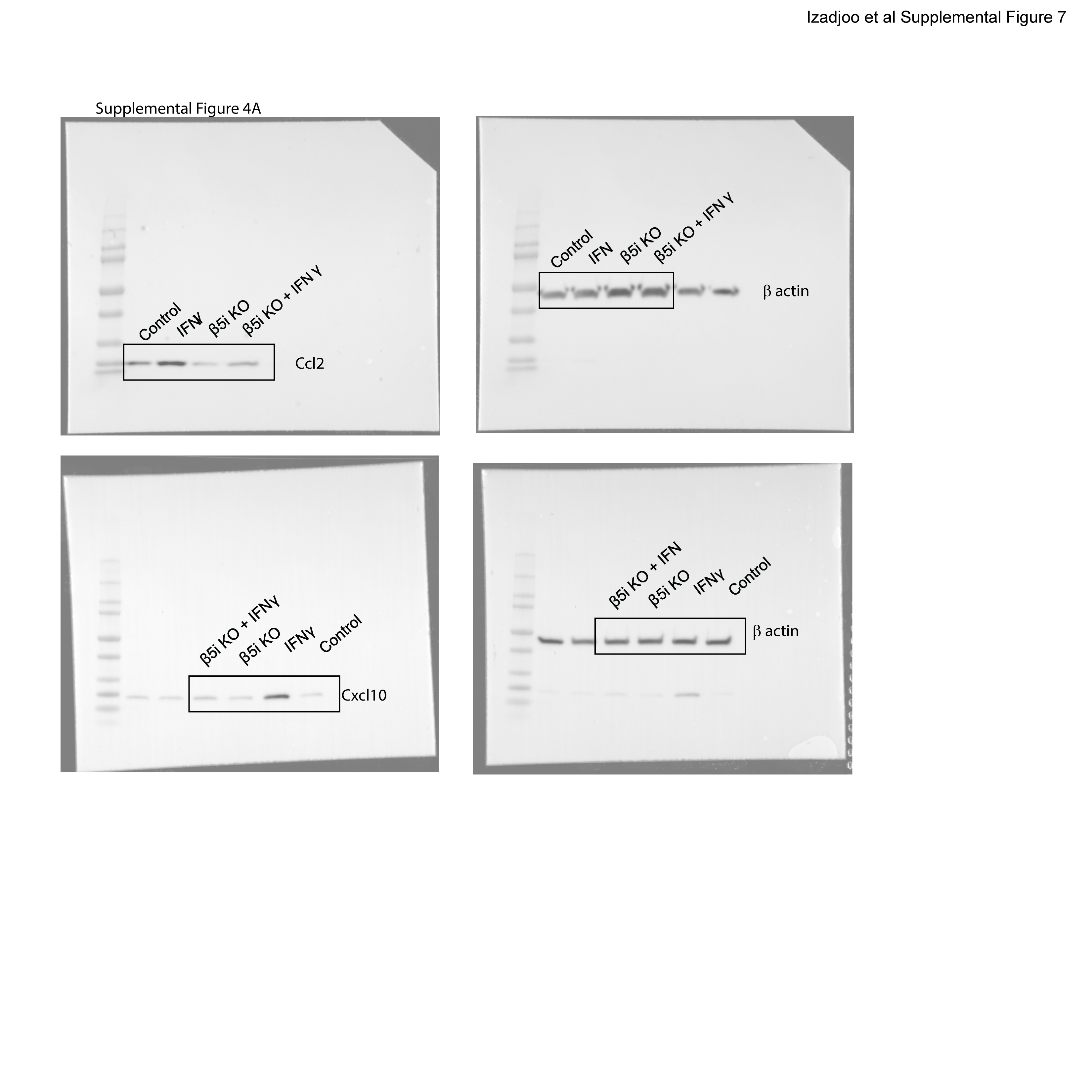

Supplement: Supplementary file 9 — Supplementary Material 9 [file 41598_2025_25341_MOESM9_ESM.tif]
